# Supplementary material for: Acceptability of HPV Vaccines: A Qualitative Systematic Review and Meta-Summary
Source: Vaccines (Basel). 2023 Sep 14;11(9):1486. doi: 10.3390/vaccines11091486 (PMC10536942; doi:10.3390/vaccines11091486)
Supplement: Supplementary file 1 [file vaccines-11-01486-s001.zip › vaccines-2509911-supplementary.pdf]

Supplementary S1: Search strategy

Electronic Databases	References Obtained
1. Medline	1265 /1384
2. The Cochrane Library	175 /290
3. Embase	1286 /1444
4. PsycINFO	149 /159
5. ERIC	47 /49
6. LILACS	68/56
7. Web of Science/SCI-EXPANDED, SSCI, A&HC	168/170
8. Conference Proceedings Citation Index-Science/Social Science & Humanities	16/16
9. Dissertations & Theses Global	137/151
10. Red de repositorios latinoamericanos (limitado por tesis)	61/62
11. Education Database	62 /74
12. Psychology Database	138/152
13. CINAHL Complete	162 /194
14. WHO ICTRP Search Portal	10 /13
15. Clinical Trials	46 /49
16. OpenGrey	21 /20
**TOTAL**	**3811/4380**

Black color: Initial search. Red color: updated

Database	: Medline
Host	: Pubmed
Date	: 2006 to February 2020

#1 "Papillomavirus Vaccines"[Mesh]

#2 ("Papillomaviridae"[Mesh]) AND (vaccine* OR vaccination OR "Vaccination"[Mesh])

#3 ("Papillomavirus Infections"[Mesh]) AND (vaccine* OR vaccination OR "Vaccination"[Mesh])

#4 ("Uterine Cervical Neoplasms"[Mesh]) AND (vaccine* OR vaccination OR "Vaccination"[Mesh])

#5 (Cervical OR cervix (neoplas* OR cancer)) AND (vaccine* OR vaccination OR "Vaccination"[Mesh])

#6 (Human Papilloma* OR HPV) AND (vaccine* OR vaccination OR "Vaccination"[Mesh])

#7 #1 OR #2 OR #3 OR #4 OR #5 OR #6

#8 (intervent* OR educat* OR communication OR advice OR advis* OR counsel* OR class OR classes)

#9 "Patient Education as Topic"[Mesh] OR "Audiovisual Aids"[Mesh] OR "Health Promotion"[Mesh]

#10 #8 OR #9

#11 "Patient Compliance"[Mesh]

#12 "Medication Adherence"[Mesh]

#13 "Patient Acceptance of Health Care"[Mesh]

#14 Adherence OR Nonadherence OR Non-adherence OR Compliance OR Noncompliance OR Non-compliance

#15 (increase* OR growth OR rise OR improve OR improv* OR promo*) AND (uptake OR initiat* OR complet* OR “informed decision-making”)

#16 #11 OR #12 OR #13 OR #14 OR #15

#17 #7 AND #10 AND #16

Search results=1.415

Database	: The Cochrane Library, CENTRAL
Host	: Wiley
Date	: 2006 to February 2020

#1 MeSH descriptor: [Papillomavirus Vaccines] explode all trees

#2 MeSH descriptor: [Papillomaviridae] explode all trees

#3 MeSH descriptor: [Vaccination] explode all trees

#4 vaccine* OR vaccination

#5 #2 AND #3

#6 #2 AND #4

#7 MeSH descriptor: [Papillomavirus Infections] explode all trees

#8 #7 AND #3

#9 #7 AND #4

#10 MeSH descriptor: [Uterine Cervical Neoplasms] explode all trees

#11 #10 AND #3

#12 #10 AND #4

#13 (Cervical OR cervix (neoplas* OR cancer))

#14 #13 AND #3

#15 #13 AND #4

#16 (Human Papilloma* OR HPV)

#17 #16 AND #3

#18 #16 AND #4

#19 #1 OR #5 OR #6 OR #8 OR #9 OR #11 OR #12 OR #14 OR #15 OR #17 OR #18

#20 (intervent* OR educat* OR communication OR advice OR advis* OR counsel* OR class OR classes)

#21 MeSH descriptor: [Patient Education as Topic] explode all trees

#22 MeSH descriptor: [Audiovisual Aids] explode all trees

#23 MeSH descriptor: [Health Promotion] explode all trees

#24 #21 OR #22 OR #23

#25 #20 OR #24

#26 MeSH descriptor: [Patient Compliance] explode all trees

#27 MeSH descriptor: [Medication Adherence] explode all trees

#28 MeSH descriptor: [Patient Acceptance of Health Care] explode all trees

#29 Adherence OR Nonadherence OR Non-adherence OR Compliance OR Noncompliance OR Non-compliance

#30 (increase* OR growth OR rise OR improve OR improv* OR promo*) AND (uptake OR initiat* OR complet* OR “informed decision-making”)

#31 #26 OR #27 OR #28 OR #29 OR #30

#32 #19 AND #25 AND #31 with Cochrane Library publication date from Jan 2006 to Jan 2019, in Trials

Search results= 290

Database	: Embase
Host	: Elsevier
Date	: 2006 to February 2020

#1 'wart virus vaccine'/exp AND [embase]/lim

#2 ('papillomaviridae'/exp) AND (vaccine* OR vaccination OR 'vaccination'/exp) AND [embase]/lim

#3 ('papillomavirus infection'/exp) AND (vaccine* OR vaccination OR 'vaccination'/exp) AND [embase]/lim

#4 ('uterine cervix tumor'/exp) AND (vaccine* OR vaccination OR 'vaccination'/exp) AND [embase]/lim

#5 (Cervical OR cervix (neoplas* OR cancer)) AND (vaccine* OR vaccination OR 'vaccination'/exp) AND [embase]/lim

#6 (Human Papilloma* OR HPV) AND (vaccine* OR vaccination OR 'vaccination'/exp) AND [embase]/lim

#7 #1 OR #2 OR #3 OR #4 OR #5 OR #6

#8 (intervent* OR educat* OR communication OR advice OR advis* OR counsel* OR class OR classes) AND [embase]/lim

#9 'patient education'/exp OR 'audiovisual aid'/exp OR 'health promotion'/exp AND [embase]/lim

#10 #8 OR #9

#11 'patient compliance'/exp AND [embase]/lim

#12 'medication compliance'/exp AND [medline]/lim

#13 'patient attitude'/exp AND [embase]/lim

#14 Adherence OR Nonadherence OR Non-adherence OR Compliance OR Noncompliance OR Non-compliance AND [embase]/lim

#15 (increase* OR growth OR rise OR improve OR improv* OR promo*) AND (uptake OR initiat* OR complet* OR “informed decision-making”) AND [embase]/lim

#16 #11 OR #12 OR #13 OR #14 OR #15

#17 #7 AND #10 AND #16

#18 #17 AND [embase]/lim AND [2006-2019]/py

Search results= 1.444

Database	: PsycINFO
Host	: EBSCO
Date	: 2006 to February 2020

S1 Papillomavirus Vaccines

S2 DE "Human Papillomavirus" AND (TI vaccine* OR TI vaccination OR AB vaccine* OR AB vaccination OR DE "Immunization")

S3 Papillomavirus Infections AND (TI vaccine* OR TI vaccination OR AB vaccine* OR AB vaccination OR DE "Immunization")

S4 Uterine Cervical Neoplasms AND (TI vaccine* OR TI vaccination OR AB vaccine* OR AB vaccination OR DE "Immunization")

S5 (Cervical OR cervix (neoplas* OR cancer)) AND (TI vaccine* OR TI vaccination OR AB vaccine* OR AB vaccination OR DE "Immunization")

S6 (Human Papilloma* OR HPV) AND (TI vaccine* OR TI vaccination OR AB vaccine* OR AB vaccination OR DE "Immunization")

S7 S1 OR S2 OR S3 OR S4 OR S5 OR S6

S8 (TI intervent* OR TI educat* OR TI communication OR TI advice OR TI advis* OR TI counsel* OR TI class OR TI classes)

S9 DE "Client Education" OR DE "Educational Audiovisual Aids" OR DE "Health Promotion"

S10 S8 OR S9

S11 DE "Treatment Compliance"

S12 Medication Adherence

S13 Patient Acceptance of Health Care

S14 Adherence OR Nonadherence OR Non-adherence OR Compliance OR Noncompliance OR Non-compliance

S15 (increase* OR growth OR rise OR improve OR improv* OR promo*) AND (uptake OR initiat* OR complet* OR “informed decision-making”)

S16 S11 OR S12 OR S13 OR S14 OR S15

S17 S7 AND S10 AND S16

Search results= 159

Database	: ERIC
Host	: OCLC
Date	: 2006 to February 2020

#1	Papillomavirus Vaccines
#2	Papillomaviridae AND (vaccine* OR vaccination OR su= "immunization programs")
#3	Papillomavirus Infections AND (vaccine* OR vaccination OR su= "immunization programs")
#4	Uterine Cervical Neoplasms AND (vaccine* OR vaccination OR su= "immunization programs")
#5	((Cervical OR cervix) AND (neoplas* OR cancer)) AND (vaccine* OR vaccination OR su= "immunization programs")
#6	(Human Papilloma* OR HPV) AND (vaccine* OR vaccination OR su= "immunization programs")
#7	#1 OR #2 OR #3 OR #4 OR #5 OR #6
#8	(intervention OR interventional OR education OR educative OR educational OR communication OR advice OR class OR classes OR advisoring OR counseling)
#9	su= "patient education" OR su= "audiovisual aids" OR su= "health promotion"
#10	#8 OR #9
#11	Patient Compliance
#12	Medication Adherence
#13	Patient Acceptance of Health Care
#14	Adherence OR Nonadherence OR Non-adherence OR Compliance OR Noncompliance OR Non-compliance
#15	(increase OR increases OR growth OR rise OR improve OR improved OR improves OR improving OR promotion OR promote OR promotes) AND (uptake OR initiation OR complete OR completion OR 'informed decision-making')
#16	#11 OR #12 OR #13 OR #14 OR #15
#17	#7 AND #10 AND #16

Search results= 73

Database	: LILACS
Host	: Bireme
Date	: 2006 to February 2020

(Papillomavirus Vaccines OR Vacunas contra Papillomavirus OR Vacinas contra Papillomavirus OR ((Papillomaviridae OR "Papillomavirus Infections" OR "Infecciones por Papillomavirus" OR "Infecções por Papillomavirus" OR "Uterine Cervical Neoplasms" OR "Neoplasias del Cuello Uterino" OR "Neoplasias do Colo do Útero" OR (Cervical OR cervix AND (neoplas$ OR cancer)) OR (Human Papilloma$ OR papiloma humano OR HPV)) AND (vaccine$ OR vacuna$ OR Vacina$ OR Vaccination OR

Vacunación OR Vacinação))) AND ((intervent$ OR educat$ OR communication OR comunicación OR comunicação OR advice OR conselhos OR consejo OR advis$ OR counsel$ OR class OR classe OR classes OR clases OR clase) OR ("Patient Education as Topic" OR "Educación del Paciente como Asunto" OR "Educação de Pacientes como Assunto" OR "Audiovisual Aids" OR "Recursos Audiovisuales" OR "Recursos Audiovisuais" OR "Health Promotion" OR "Promoción de la Salud" OR "Promoção da Saúde")) AND (("Patient Compliance" OR "Cooperación del Paciente" OR "Cooperação do Paciente" OR "Medication Adherence" OR "Cumplimiento de la Medicación" OR "Adesão à Medicação" OR "Patient Acceptance of Health Care" OR "Aceptación de la Atención de Salud" OR "Aceitação pelo Paciente de Cuidados de Saúde") OR (Adherence OR Adherencia OR Adesão OR Nonadherence OR Não-adesão OR No-adherencia OR Non-adherence OR Compliance OR conformidad OR Conformidade OR Noncompliance OR Non-compliance OR disconforme OR Não-conformidade) OR ((increase$ OR growth OR crecimiento OR crescimento OR rise OR melhorar OR mejorar OR improve OR improv$ OR promo$) AND (uptake OR initiative OR initiation OR initiating OR complet$ OR iniciativa OR incrementa OR iniciação OR 'informed decision-making' OR "decisiones informadas" OR "decisões informadas"))) OR ((HPV OR VPH OR papilloma) AND (vaccine$ OR vacuna$ OR preven$)) AND (intervent$ OR intervenc$)

Search results= 68

Database	: Web of Science
Host	: Clarivate Analytics
Date	: 2006 to February 2020

#1 (TS= Papilloma* OR TI=papilloma*) AND (TS= vaccine* OR TS= vaccination OR TI=vaccine* OR TI= vaccination)

#2 (TS= Uterine Cervical Neoplasm* OR TI= Uterine Cervical Neoplasm*) AND (TS= vaccine* OR TS= vaccination OR TI=vaccine* OR TI= vaccination)

#3 ((TI= Cervical OR TI= cervix OR TS= Cervical OR TS= cervix) AND (TI= neoplas* OR TI= cancer OR TS= neoplas* OR TS= cancer)) AND (TS= vaccine* OR TS= vaccination OR TI=vaccine* OR TI= vaccination)

#4 (TI=HPV OR TS=HPV) AND (TS= vaccine* OR TS= vaccination OR TI=vaccine* OR TI= vaccination)

#5 #1 OR #2 OR #3 OR #4

#6 (TI= intervent* OR TI= educat* OR TI= communication OR TI= advice OR TI= advis* OR TI= counsel* OR TI= class OR TI= classes)

#7 TS="Audiovisual aids" OR TS="Audiovisual aids" OR TS="Health promotion" OR TI="Health promotion"

#8 #6 OR #7

#9 (TS= "Patient Compliance" OR TS= "Patient Acceptance of Health Care" OR TI= "Patient Compliance" OR TI= "Patient Acceptance of Health Care")

#10 (TI= Adherence OR TI= Nonadherence OR TI= Non-adherence OR TI= Compliance OR TI= Noncompliance OR TI= Non-compliance OR TS= Adherence OR TS= Nonadherence OR TS= Non-adherence OR TS= Compliance OR TS= Noncompliance OR TS= Non-compliance)

#11 (TI= increase* OR TI= growth OR TI= rise OR TI= improve OR TI= improv* OR TI= promo* OR TS= increase* OR TS= growth OR TS= rise OR TS= improve OR TS= improv* OR TS= promo*) AND (TI= uptake OR TI= initiat* OR TI= complet* OR TI= “informed decision*” OR TS= uptake OR TS= initiat* OR TS= complet* OR TS= “informed decision*”)

#12 #9 OR #10 OR #11

#13 #5 AND #8 AND #12

Search results= 173

Database : Conference Proceedings Citation Index-Science/ Conference Proceedings Citation Index-Social Science & Humanities
Host : Clarivate Analytics
Date : 2006 to February 2020

#1 (TS= Papilloma* OR TI=papilloma*) AND (TS= vaccine* OR TS= vaccination OR TI=vaccine* OR TI= vaccination)

#2 (TS= Uterine Cervical Neoplasm* OR TI= Uterine Cervical Neoplasm*) AND (TS= vaccine* OR TS= vaccination OR TI=vaccine* OR TI= vaccination)

#3 ((TI= Cervical OR TI= cervix OR TS= Cervical OR TS= cervix) AND (TI= neoplas* OR TI= cancer OR TS= neoplas* OR TS= cancer)) AND (TS= vaccine* OR TS= vaccination OR TI=vaccine* OR TI= vaccination)

#4 (TI=HPV OR TS=HPV) AND (TS= vaccine* OR TS= vaccination OR TI=vaccine* OR TI= vaccination)

#5 #1 OR #2 OR #3 OR #4

#6 (TI= intervent* OR TI= educat* OR TI= communication OR TI= advice OR TI= advis* OR TI= counsel* OR TI= class OR TI= clases OR TS= intervent* OR TS= educat* OR TS= communication OR TS= advice OR TS= advis* OR TS= counsel* OR TS= class OR TS= classes)

#7 TS="Audiovisual aids" OR TS="Audiovisual aids" OR TS="Health promotion" OR TI="Health promotion"

#8 #6 OR #7

#9 (TS= "Patient Compliance" OR TS= "Patient Acceptance of Health Care" OR TI= "Patient Compliance" OR TI= "Patient Acceptance of Health Care")

#10 (TI= Adherence OR TI= Nonadherence OR TI= Non-adherence OR TI= Compliance OR TI= Noncompliance OR TI= Non-compliance OR TS= Adherence OR TS= Nonadherence OR TS= Non-adherence OR TS= Compliance OR TS= Noncompliance OR TS= Non-compliance)

#11 (TI= increase* OR TI= growth OR TI= rise OR TI= improve OR TI= improv* OR TI= promo* OR TS= increase* OR TS= growth OR TS= rise OR TS= improve OR TS= improv* OR TS= promo*) AND (TI= uptake OR TI= initiat* OR TI= complet* OR TI= “informed decision*” OR TS= uptake OR TS= initiat* OR TS= complet* OR TS= “informed decision*”)

#12 #9 OR #10 OR #11

#13 #5 AND #8 AND #12

Search results= 17

Database	: Dissertations & Theses Global
Host	: ProQuest
Date	: 2006 to February 2020

ti(HPV OR "human papillomavirus" vaccin*) AND ft(intervent* OR educat* OR communication OR advice OR advis* OR counsel* OR class OR classes) AND ft(Adherence OR Nonadherence OR Non-adherence OR Compliance OR Noncompliance OR Non-compliance)

Limited by Doctoral dissertations

Search results=168

Database	: Red de repositorios latinoamericanos
Host	: Universidad de Chile
Date	: 2006 to February 2020

(hpv OR vph OR papilloma OR papiloma OR papilomavirus OR papillomavirus) AND (vacuna OR vaccine OR vacunas OR vaccination OR vacines OR vacunación OR vacinas OR Vacinação)

Limited by Thesis

Search results=64

Database	: Education Database
Host	: ProQuest
Date	: 2006 to February 2020

ti(HPV OR "human papillomavirus" vaccin*) AND ft(intervent* OR educat* OR communication OR advice OR advis* OR counsel* OR class OR classes) AND ft(Adherence OR Nonadherence OR Non-adherence OR Compliance OR Noncompliance OR Non-compliance)

Search results=74

Database	: Psychology Database
Host	: ProQuest
Date	: 2006 to February 2020

ti(HPV OR "human papillomavirus" vaccin*) AND ft(intervent* OR educat* OR communication OR advice OR advis* OR counsel* OR class OR classes) AND ft(Adherence OR Nonadherence OR Non-adherence OR Compliance OR Noncompliance OR Non-compliance)

Search results=138

Database	: CINAHL Complete
Host	: EBSCO
Date	: 2006 to February 2020

S1	(MH "Papillomavirus Vaccine")
S2	(MH "Papillomaviruses") AND (vaccine* OR vaccination OR (MH "Immunization+"))
S3	(MH "Papillomavirus Infections+") AND (vaccine* OR vaccination OR (MH "Immunization+"))
S4	(MH "Cervix Neoplasms+") AND (vaccine* OR vaccination OR (MH "Immunization+"))
S5	(Cervical OR cervix (neoplas* OR cancer)) AND (vaccine* OR vaccination OR (MH "Immunization+"))
S6	(Human Papilloma* OR HPV) AND (vaccine* OR vaccination OR (MH "Immunization+"))
S7	S1 OR S2 OR S3 OR S4 OR S5 OR S6
S8	(TI intervent* OR TI educat* OR TI communication OR TI advice OR TI advis* OR TI counsel* OR TI class OR TI classes)
S9	(MH "Patient Education+") OR (MH "Audiovisuals+") OR (MH "Health Promotion+")
S10	S8 OR S9
S11	(MH "Patient Compliance+")
S12	(MH "Medication Compliance")
S13	Patient Acceptance of Health Care
S14	Adherence OR Nonadherence OR Non-adherence OR Compliance OR Noncompliance OR Non-compliance
S15	(increase* OR growth OR rise OR improve OR improv* OR promo*) AND (uptake OR initiat* OR complet* OR “informed decision-making”)
S16	S11 OR S12 OR S13 OR S14 OR S15
S17	S7 AND S10 AND S16

Search results=162

Database	: WHO ICTRP Search Portal
Host	: http://apps.who.int/trialsearch
Date	: 2006 to February 2020

Advanced search (with Recruitment set at ALL):

Search 1

Condition: papillomavirus OR HPV OR papillomaviridae OR human papilloma

Intervention: (vaccine OR vaccination) AND (education OR adherence OR non-adherence OR advise OR advice)

Search results= 10 trials

Database	: Clinical Trials
Host	: https://clinicaltrials.gov
Date	: 2006 to February 2020

Advanced search (with Recruitment status set at all studies)

Search

Condition or disease: papillomavirus OR HPV OR papillomaviridae OR human papilloma

Study type: Interventional Studies (Clinical Trials)

Study results: All studies

Targeted Search

Intervention/treatment: vaccine OR vaccines OR vaccination

Title / Acronym: education OR adherence OR non-adherence OR advise OR advice OR intervention OR compliance

Search results= 46

Database	: OpenGrey
Host	: http://www.opengrey.eu/
Date	: 2006 to February 2020

(HPV OR papilloma* OR cervi* cancer) AND (vaccin* OR Immunization*) AND (intervent* OR educat* OR communicat* OR advice OR advis* OR counsel* OR class OR classes)

Search results=21
